# Treatment outcomes of stereotactic ablative body radiotherapy on oligometastases from colorectal cancer: early results of a single institution service evaluation

**DOI:** 10.1259/bjro.20210071

**Published:** 2022-03-11

**Authors:** Julie Duong, Adele Stewart-Lord, Prasana Nariyangadu, Mark Harrison, Yat Man Tsang

**Affiliations:** ^1^ Mount Vernon Cancer Centre, Northwood, Middlesex HA6 2RN, UK; ^2^ School of Health and Social Care, London Southbank University, London, UK

## Abstract

**Objective:**

Stereotactic ablative radiotherapy (SABR) has been suggested to be an effective non-invasive ablative therapy for oligometastases originated from colorectal cancer (CRC). This study aimed to report CRC oligometastases SABR treatment outcomes in terms of overall survival (OS), progression-free survival (PFS) and post-treatment toxicities.

**Methods:**

Treatment records of patients with CRC metachronous oligometastases who underwent SABR at a single institution between February 2015 and December 2018 were retrospectively reviewed. OS and PFS were calculated using Kaplan–Meier statistics and post-RT toxicity data was scored following CTCAE v. 4.0. Analysis of prognostic factors on OS and PFS was performed based on site of primary cancer, types of treatment to primary cancer, number of oligometastases, SABR treatment sites, intervals between treatment to primary cancer and SABR to oligometastases, biological equivalent dose, cumulative gross tumour volume and planning target volume.

**Results:**

75 patients with 86 CRC metachronous oligometastases (including liver, lung, lymph nodes and bone) were included. The median age was 65.5 years (range 42.5–87.2) with a median follow-up of 23.8 months (range 3.1–46.5). The estimated median PFS was 14.6 months (95% CI 9.6–19.6). and estimated median OS was 33.3 months (95% CI 22.9–43.7). Majority of patients tolerated SABR well with the most common acute side-effects of Grade 1 fatigue. No Grade 3 or higher toxicities were reported at any time points.

Only SABR treatment sites (*p* = 0.03) and cumulative volumes of planning target volume (*p* = 0.02) were found to be statistically significant independent predictors of PFS and OS respectively.

**Conclusion:**

This study showed modest PFS, OS, and post-treatment toxicity outcomes on SABR to metachronous oligometastases from CRC. It has highlighted that cumulative tumour volume may be a stronger prognostic factor of OS comparing to the number of metastases.

**Advances in knowledge:**

There are limited data published on the efficacy and post-treatment toxicity of CRC oligometastases SABR with adequate length of follow-up. Our retrospective study suggests that cumulative tumour volume may be a stronger prognostic factor of OS comparing to the number of oligometastases.

## Background

The concept of oligometastases refers to a clinical state of a limited number of detectable metastatic tumours.^1^ At the time of diagnosis, approximately 25% of colorectal cancer (CRC) patients present with a solitary metastasis or oligometastases. The liver and lungs are the common sites of metastases for CRC where 60–71% of the CRC metastases occur in the liver and 25–40% occur in the lungs.^2,3^ Other sites of CRC metastases include the lymph nodes (16%), bones (5–10%), ovaries (3–5%) and 1% in the adrenal glands and central nervous systems.^3,4^

Most patients presenting with CRC oligometastases are typically treated with surgery and adjuvant/neoadjuvant chemotherapy which leads to improvements in local control and survival rates.^5,6^ Despite the benefits with surgical resection, approximately 80% of patients with oligometastases are not suitable candidates for curative surgical resection due to medical contraindications, locations, numbers and sizes of the metastases.^7,8^

For medically inoperable metastases, there is emerging evidence of using stereotactic ablative radiotherapy (SABR) for oligometastases.^9^ SABR is deemed to be an effective non-invasive ablative therapy in the treatment of oligometastases (including liver, lung, lymph nodes and bone) offering promising local control rates and acceptable post treatment toxicities.^10–13^ Currently, the majority of outcome data for oligometastases treated with SABR are from primary sites of prostate and breast cancer histology.^12,13^ Concerns are raised to question whether CRC oligometastases may represent an inherently more radioresistant histology.^14^ There are limited outcome data for multisite oligometastases from CRC treated with SABR, in order to identify potential factors associated with treatment outcomes in this CRC cohort.^14,15^

Against this background, this study aimed to investigate the factors influencing SABR treatment outcomes in terms of overall survival (OS), progression-free survival (PFS) and post-treatment toxicity in patients receiving SABR for oligometastases originated from CRC.

## Methods and materials

All patients receiving SABR for oligometastases originated from CRC at our institution were eligible for this study. They were with confirmed histological diagnosis, WHO performance status 0–2, no more than three sites of metachronous metastatic disease, maximum size of 6 cm for any single metastasis, and expected life expectancy greater than 6 months as specific inclusion criteria for the study. Patients with brain metastases or less than 3 months of follow-up were excluded. This work was undertaken as a single institution service evaluation under the approval of the institutional research ethics board. All patients provided written informed consent for their data to be used in this study.

SABR was performed with daily image-guided radiotherapy (IGRT) using a dedicated robotic stereotactic radiotherapy machine - CyberKnife (Accuray Inc., Sunnyvale, CA). All patients having SABR were immobilised with an individualised vacuum cushion or a thermoplastic shell and they were scanned with helical CT using 1.5 mm interval. The gross tumour volume (GTV) was identified on the CT scans and considered equal to the clinical target volume (CTV) for all lesions. The planning target volume (PTV) was defined as the GTV plus a set-up margin. 2 mm PTV margin was used for spine lesions and 3–5 mm PTV margin was used in node and bone lesions. Where disease sites were subject to internal movement (such as lung or liver), patients were planned using four-dimensional (4D) CT scans. Fiducial tracking was used for abdominal motion management. A uniform PTV expansion of 5–10 mm margin was used in lung and liver cases

**Figure 1.**
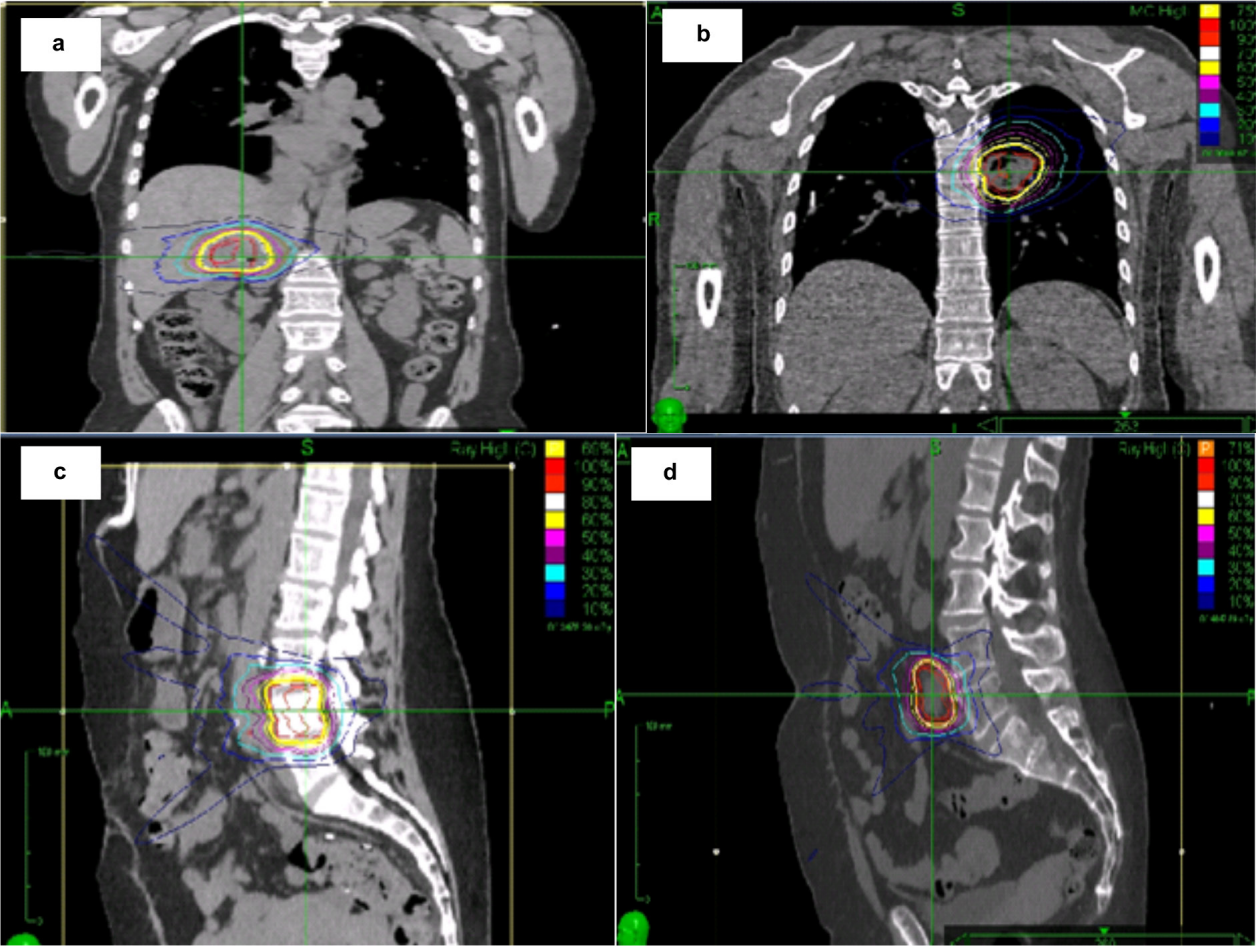
Dose distribution of SABR plans for CRC patients with oligometastases in liver (Figure 1a), lung (Figure 1b), bone (Figure 1c), and lymph node (Figure 1d). CRC, colorectal cancer; SABR, stereotactic ablative radiotherapy.

Radiation doses varied depending on lesion site as per institutional policy: 50–55 Gy in 3–5 fractions for lung tumours, 40–50 Gy in 3–5 fraction for liver tumours, 24–30 Gy in 3 fractions for spine/bone tumours and 27–35 Gy in 3–5 fractions for nodal tumours. Typical SABR plan dose distributions for the treatment sites are shown in Figure 1. An a/b ratio of 10 was used for biologically effective dose (BED_10_) calculations.

Follow-up visits included clinical evaluation and diagnostic imaging (CT, MRI or positron emission tomography scan) at treating clinician’s discretion. Assessments were performed at baseline, 1, 3, and 6 months after SABR, then at 6 monthly intervals thereafter and imaging at the same time points. Acute toxicity was defined as that occurring within 3 months days post-SABR; all reported toxicity thereafter was classified as late toxicity. Toxicity was recorded according to the National Cancer Institute Common Terminology Criteria for Adverse Events v. 4.0.

The primary outcome measures were the treatment outcomes in terms of PFS and OS. PFS was defined as the absence of any progression (local, regional, or distant) and calculated as time from the start of SABR to disease progression at any site or to the last date of follow-up if no progression occurred. OS was calculated from the start of SABR to death from any cause or last date of follow-up. PFS and OS rates for the whole population were calculated using the Kaplan–Meier method.^14^ The patient subgroups of the site of primary cancer, type of treatments to primary cancer, number of oligometastases, SABR treatment sites, intervals between treatment to primary cancer and SABR to oligometastases (INT), BED_10_, cumulative volumes of GTV and PTV were defined as co-variates. For INT, BED_10_, GTV and PTV volumes, the median of the whole cohort was used to separate the patients into two groups: ≤median and >median. Univariate Cox regression analysis was performed to determine if any of these co-variates predicted for PFS and OS.^14^ All the variables with a *p*-value of <0.10 were entered into a multivariate, forward conditional Cox regression. For all tests, a *p*-value of <0.05 was considered statistically significant. Statistical analysis was performed with SPSS v. 26.0 (IBM Corp., Armonk, NY).

## Results

In between February 2015 and December 2018, 75 patients with 86 metachronous oligometastases from CRC were treated with SABR. The median patient age was 65.5 years (range 42.5–87.2 years) with a median follow-up of 23.8 months (range 3.1–46.5 months). Table 1 summarises the baseline clinical- and treatment-related parameters for the entire cohort. Majority of the cases were lymph nodes (61.3%), followed by livers (26.7%), lungs (6.7%) and bones (5.3%). The SABR fractionation schedules were either 3 (47%) or 5 (53%) fractions. The median BED_10_ of the whole cohort was 60 Gy (range 37.5–151.2 Gy) while the median BED_10_ of the patients with nodal oligometastases was 55.3 Gy (range 37.5–79.2 Gy).

**Table 1.** Baseline and treatment-related patient characteristics

Parameter	Number (%)
Patients (n)	75
Age (years)	
Median	65.5
Range	42.5–87.2
Site of primary cancer	
Colon	36 (48%)
Rectum	39 (52%)
Type of treatments to primary cancer	
Surgery + neoadjuvant chemo	40 (53%)
Other	35 (47%)
Previous radiotherapy to primary cancer	
Yes	21 (28%)
No	54 (72%)
Previous chemotherapy to primary cancer	
Yes	63 (84%)
No	12 (15%)
Number of oligometastases	
1	65 (87%)
2–3	10 (13%)
SABR treatment sites	
Node	46 (61%)
Non-node (liver, lung and bone)	29 (39%)
Intervals between treatment to primary cancer and SABR to oligometastases (median = 32 months)	
≤32 months	38 (51%)
>32 months	37 (49%)
BED_10_ (median = 60 Gy)	
≤60 Gy	32 (43%)
>60 Gy	43 (57%)
Cumulative volumes of GTV (median = 17.6 cc)	
≤17.6 cc	38 (51%)
>17.6 cc	37 (49%)
Cumulative volumes of PTV (median = 43.4 cc)	
≤43.4 cc	38 (51%)
>43.4 cc	37 (49%)

BED, biologically effective dose; GTV, gross tumour volume; PTV, planning target volume; SABR, stereotactic ablative radiotherapy.

**Figure 2.**
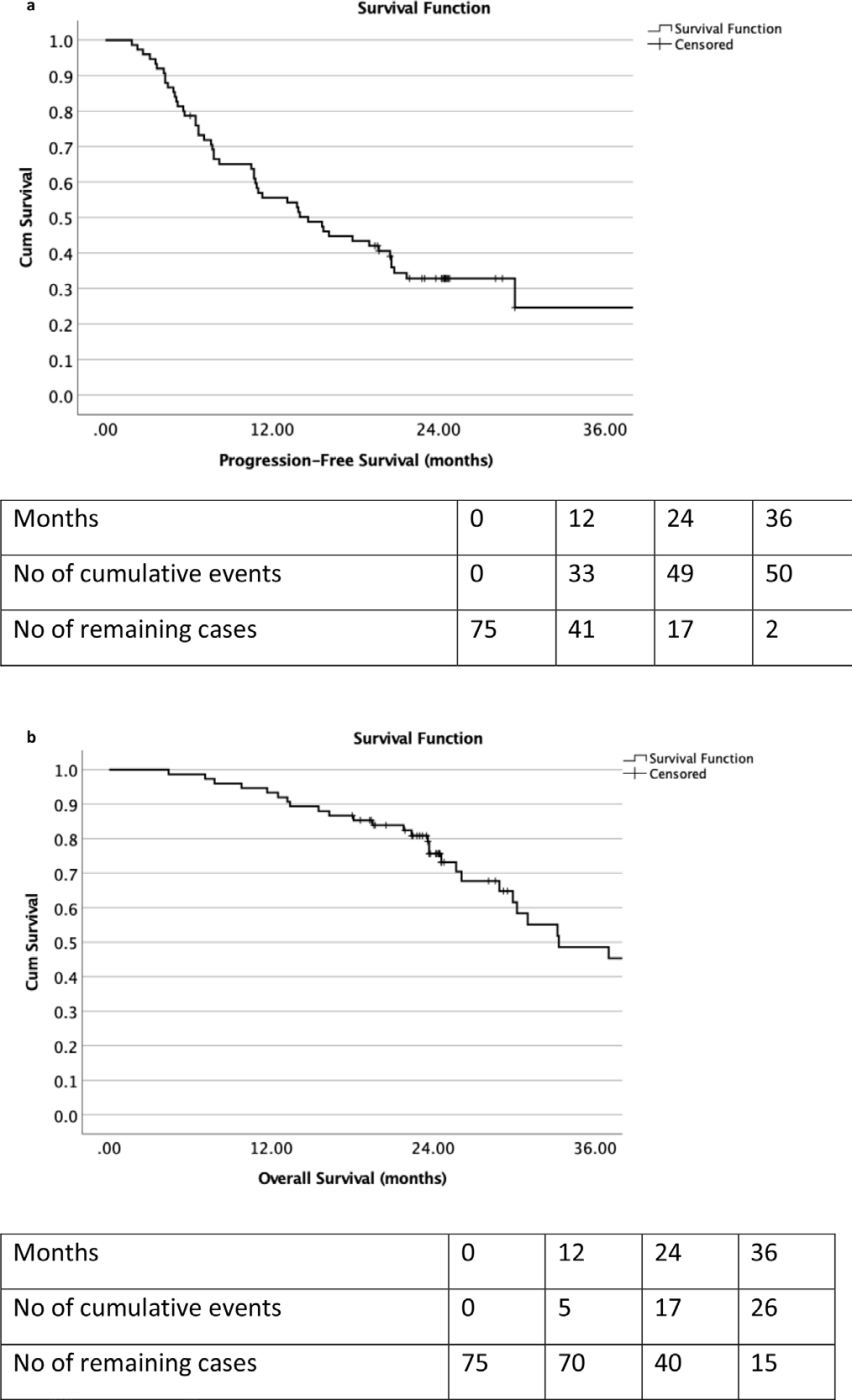
Kaplan–Meier progression free survival (Figure 2a) and overall survival (Figure 2b) curves for all CRC patients with oligometastases using stereotactic ablative body radiotherapy. CRC, colorectal cancer.

As illustrated in Figure 2, the estimated median PFS was 14.6 months (95% CI 9.6–19.6 months). The PFS at 1 year and 2 years were 55.6% and 32.8% respectively. The estimated median OS was 33.3 months (95% CI 22.9–43.7 months). The OS at 1 year and 2 years were 93.3% and 75.6% respectively.

As illustrated in Table 2, the vast majority of patient tolerated the treatment well with the most common acute side-effects of Grade 1 fatigue reported (31%), followed by genitourinary (8.0%), back pain (5.3%) and gastrointestinal (4.0%). No Grade 3 or higher toxicities were reported at any time points.

**Table 2.** Acute and late toxicities after stereotactic ablative body radiotherapy using CTCAE v. 4.0

	Acute (≤6 months)			Late (>6 months)		
Toxicity event	4–6 weeks	3 months	6 months	12 months	18 months	24 months
Fatigue n (%)	24 (32%)	22 (29.3%)	22 (29.3%)	22 (29.3%)	20 (26.7%)	13 (17.3%)
Grade 1	23 (31%)	22 (29.3%)	22 (29.3%)	22 (29.3%)	20 (26.7%)	13 (17.3%)
Grade 2	1 (1.3%)	0	0	0	0	0
Nausea n (%)	2 (2.7%)	1 (1.3%)	1 (1.3%)	0	0	0
Grade 1	2 (2.7%)	1 (1.3%)	1 (1.3%)			
Grade 2	0	0	0			
Cough n (%)	1 (1.3%)	1 (1.3%)	1 (1.3%)	0	0	0
Grade 1	1 (1.3%)	1 (1.3%)	1 (1.3%)			
Grade 2	0	0	0			
Spinal fracture						
n (%)	4 (5.3%)	3 (4%)	3 (4%)	3 (4%)	2 (2.7%)	1 (1.3%)
Grade 1	2 (2.7%)	2 (2.7%)	2 (2.7%)	2 (2.7%)	1 (1.3%)	0 (0%)
Grade 2	2 (2.7%)	1 (1.3%)	1 (1.3%)	1 (1.3%)	1 (1.3%)	1 (1.3%)
Genitourinary:						
n (%)	6 (8%)	6 (8%)	5 (6.7%)	5 (6.7%)	3 (4%)	4 (5.3%)
Grade 1	6 (8%)	6 (8%)	5 (6.7%)	5 (6.7%)	3 (4%)	4 (5.3%)
Grade 2	0	0	0	0	0	0
Gastrointestinal:						
n (%)	3 (4%)	2 (2.7%)	2 (2.7%)	1 (1.3%)	3 (4%)	3 (4%)
Grade 1	2 (2.7%)	1 (1.3%)	1 (1.3%)	0	3 (4%)	3 (4%)
Grade 2	1 (1.3%)	1 (1.3%)	1 (1.3%)	1 (1.3%)	0	0

CTCAE, Common Terminology Criteria for Adverse Events.

n represents the number of patients. Genitourinary toxicities refer to urinary urgency, urinary frequency, urinary incontinence, and urinary retention. Gastrointestinal toxicities refer to proctitis, diarrhea and haemorrhage.

Proportional hazard ratios (HRs) and significance levels for uni- and multivariate Cox regression analyses on PFS and OS are listed in Table 3. The SABR treatment sites and status of previous radiotherapy to primary cancer were statistically significant factors (*p* < 0.10) for PFS in the univariate Cox regression analysis. The cumulative volumes of GTV and PTV were statistically significant factors for OS. These were entered into a multivariate, forward conditional Cox regression test. After adjustment, only SABR treatment sites (*p* = 0.03) and cumulative volumes of PTV (*p* = 0.02) were found to be statistically significant independent predictors of PFS and OS respectively.

**Table 3.** Univariate and multivariate analyses for whole study population showing prognostic factors of PFS and OS

Variables for PFS	*Univariate*			*Multivariate*		
	HR	95% CI	*p*	HR	95% CI	*p*
Site of primary cancer Colon *vs* Rectum	0.87	0.50–1.53	0.63			
Type of treatments to primary cancer Surgery + neoadjuvant chemo *vs* other	0.64	0.37–1.13	0.12			
Previous radiotherapy Yes *vs* No	1.69	0.94–3.03	0.08			0.14
Previous chemotherapy Yes *vs* No	1.40	0.60–3.30	0.44			
Number of oligometastases one *vs* 2–3	0.80	0.36–1.79	0.59			
SABR treatment sites Node *vs* Non-node	0.54	0.31–0.94	0.03	0.54	0.31–0.94	0.03
Intervals between treatment to primary cancer and SABR to oligometastases ≤32 months vs >32 months	1.23	0.71–2.14	0.47			
BED_10_ ≤60 Gy vs >60 Gy	0.75	0.43–1.32	0.32			
Cumulative volumes of GTV ≤17.6 cc vs >17.6 cc	0.83	0.48–1.44	0.50			
Cumulative volumes of PTV ≤43.4 cc vs >43.4 cc	0.91	0.52–1.57	0.73			
**Variables for OS**	*Univariate*			*Multivariate*		
	HR	95% CI	*p*	HR	95% CI	*p*
Site of primary cancer Colon *vs* Rectum	0.92	0.48–1.79	0.81			
Type of treatments to primary cancer Surgery + neoadjuvant chemo *vs* other	1.39	0.71–2.69	0.34			
Previous radiotherapy Yes *vs* No	0.95	0.47–1.90	0.88			
Previous chemotherapy Yes *vs* No	2.19	0.66–7.27	0.20			
Number of oligometastases one *vs* 2–3	1.11	0.39–3.21	0.84			
SABR treatment sites Node *vs* Non-node	0.67	0.35–1.28	0.22			
Intervals between treatment to primary cancer and SABR to oligometastases ≤32 months vs >32 months	1.33	0.69–2.53	0.39			
BED_10_ ≤60 Gy vs >60 Gy	0.92	0.48–1.76	0.79			
Cumulative volumes of GTV ≤17.6 cc vs >17.6 cc	0.45	0.22–0.92	0.03			0.38
Cumulative volumes of PTV ≤43.4 vs 43.4 cc	0.45	0.22–0.89	0.02	0.45	0.22–0.89	0.02

BED, biologically effective dose; CI, confidenec interval; OS, overall survival; PFS, progression-free survival; GTV, gross tumour volume; HR, hazard ratio; PTV, planning target volume; SABR, stereotactic ablative radiotherapy.

**Figure 3.**
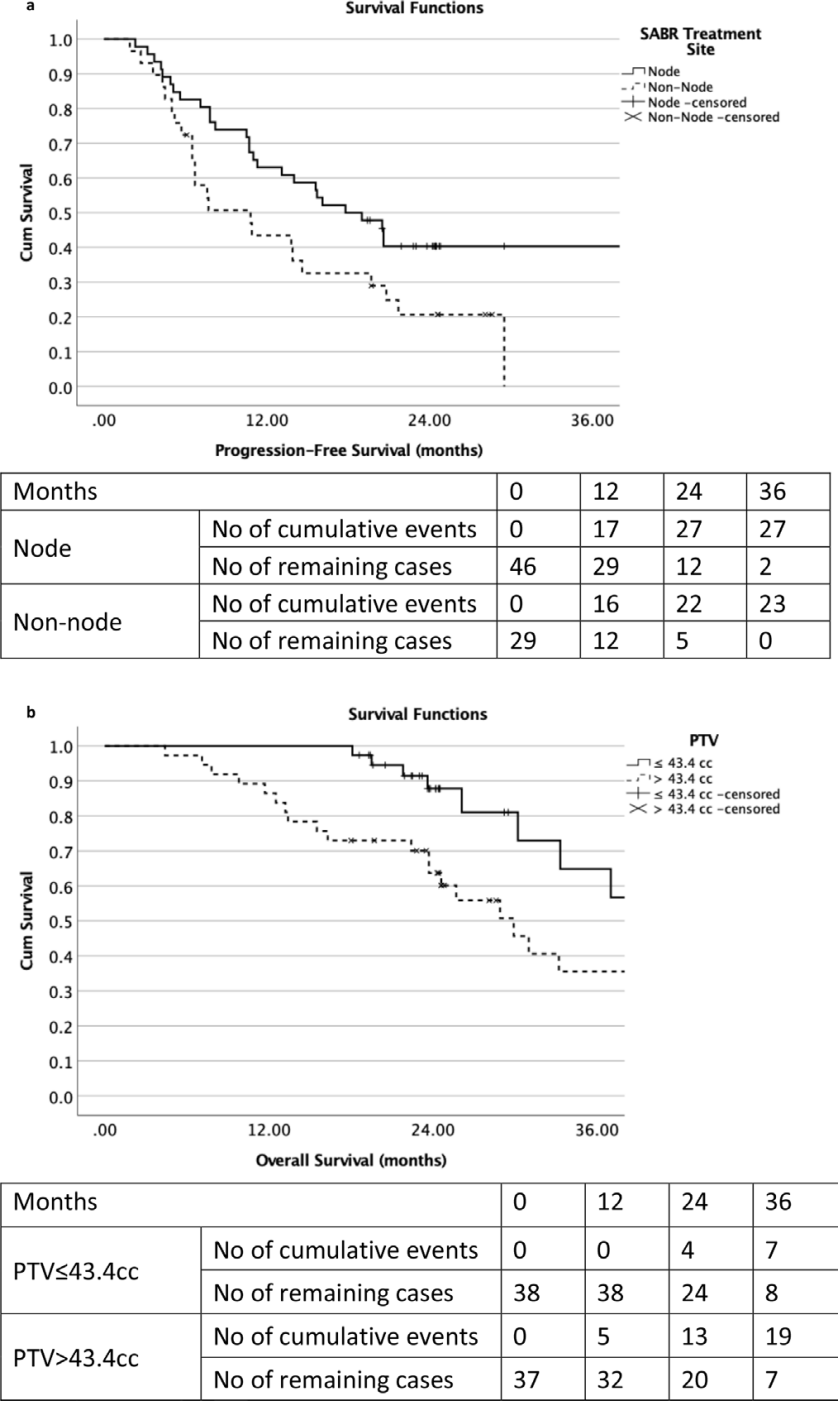
Kaplan–Meier progression-free survival curves for the node and non-node SABR treatment site groups (Figure 3a); and overall survival curves for the cumulative volumes of PTV ≤43.4 cc and >43.4 cc groups (Figure 3b). PTV, planning target volume; SABR, stereotactic ablative radiotherapy.

As illustrated in Figure 3, the estimated median PFS of the node SABR treatment site group was 17.8 months (95% CI 13.3–22.3 months) compared to 10.8 months (95% CI 5.4–16.2 months) of the non-node group. The estimated median OS of the PTV ≤ 43.4 cc group was 42.5 months (95% CI 32.0–53.0 months) compared to 29.9 months (95% CI 23.3–36.5 months) of the PTV > 43.4 cc group.

## Discussion

SABR is a modern high-precision external-beam radiotherapy technique where the tumour is targeted accurately and precisely with a high dose of radiation and rapid dose fall-off gradients.^16^ One of the main goals of delivering SABR to patients with oligometastases is to achieve long-term control of each oligometastatic site with the aim of translating this into potential survival benefits.

Within our cohort, the estimated PFS and OS at 1 year were 55.6 and 93.3% respectively. These were comparable with the findings reported in the systematic reviews by Kobiela et al^12^ and Petrelli et al.^17^ This study confirmed that SABR was a safe, non-invasive treatment option to patients with metachronous oligometastases from CRC in terms of the acceptable post-treatment toxicities (no Grade 3 toxicities reported).

CRC patients with oligometastatic disease were often deemed to have worse treatment outcomes compared to oligometastatic disease from other primary sites when treated with SABR.^13^ Most studies investigating SABR on oligometastases from CRC usually reported on the SABR sites of lung and liver.^12,18,19^ In our study, lymph nodes were the most common treated site of oligometastases. Typically, surgical resection on lymph nodes can be challenging when there are major vascular structures nearby, the tumour invades nearby critical organs such as the pancreas or bile duct and lastly, the patient has poor performance or comorbidities.^20^ The potential risk for gastrointestinal toxicities can sometimes limit the role of radiotherapy. Two severe Grade 4 complications were reported in lymph node SABR cohort by Kang et al.^21^ This could be possibly explained by the fact that the SABR prescription dose was as high as 48–51 Gy in 3 fractions (BED_10_ up to 137.7 Gy) delivered to the lymph nodes. Intestinal obstruction occurred with 48 Gy to a large lymph node (PTV size was 40 cc) and rectal perforation occurred in the lymph node treated with 51 Gy.^21^ A range of SABR fractionation schedules were used in this study and they were in agreements with the ones reported in the SABR-COMET trial.^22^ SABR fractionation schedules can be varied based on the anatomic treatment site and how close the organs at risk are located to the tumours. Supported by studies by Thompson et al^15^ and Comito et al,^19^ our study suggested that BED_10_ was not a statistically significant prognostic factor for PFS and OS. Improved local control rates were often reported to be associated with higher BED_10_ (>100 Gy) in SABR to lung and liver oligometastases.^9,23,24^ In our study cohort, the median BED_10_ was 55.3 Gy (range 37.5–79.2 Gy) of patients with nodal oligometastases and there was no Grade 3 or above post-treatment toxicity reported. These implied that each SABR treatment site should have its own specific fractionation schedule and extra caution should be taken in terms of post-treatment toxicities with SABR treatments of high BED_10_. In agreement with O’Cathail et al,^25^ the estimated median PFS of our whole cohort was 14.6 months and the multivariate Cox regression analysis suggested that CRC patients with nodal oligometastatic disease had better PFS compared to non-nodal oligometastatic disease (median 17.8 vs 10.8 months).

There was a limited number of studies investigating the prognostic factors of SABR treatment outcomes in patients with oligometastases from CRC. The treatment management to the primary cancer (with or without radiotherapy) was observed to influence PFS in the univariate analysis. Types of treatment to the primary cancer were not commonly examined as a factor on PFS in CRC oligometastases when treated with SABR due to the complex management of metastatic CRC. Published literature usually aims to examine the influence of previous systemic treatment prior to SABR on treatment outcomes, in which this was not a significant factor influencing PFS and OS in this study. This study is one of the first to highlight that patients who had an initial radiotherapy to their primary cancer may have worse PFS than patients who had no radiotherapy in the management of their primary cancer. One of the possible explanations for this is that any oligometastases subsequently developed post radiotherapy to the primary cancer can be relatively radioresistant, provided that all the CRC oligometastases being metachronous in this study. Further studies with a bigger sample size are needed to investigate the impact of this factor.

Both cumulative GTV and PTV volumes were statistically significant prognostic factors on OS in the univariate Cox regression analysis while the cumulative PTV volumes remained as a statistically significant factor on predicting OS in the multivariate analysis. There was an estimated improvement in median OS of 12 months when comparing the groups of PTV ≤ 43.4 cc and >43.4 cc. Patients with smaller tumour volumes were often associated with better OS.^26–29^ Thompson et al^14^ reported that PTV volumes had significant impact on OS. The median cumulative PTV within the study by Thompson et al^15^ was 50 cc which was comparable to ours. This implied that the cumulative PTV volumes could be a better prognostic factor on OS and should be used as a criterion in selecting patients for SABR before widespread disease occurs as opposed to the total number of oligometastases.

It is acknowledged that the obvious limitations of our study are the relatively small sample size and short follow-up period. This has made it difficult to carry out post-hoc subgroup analysis especially for the lymph nodes oligometastases to further strengthen the study results. Due to its retrospective nature as a service evaluation, no local control outcome data post-SABR were available and there were demographic differences in the patient subgroup analysis. KRAS mutation has been suggested as an important predicator to resistance of antiepidermal growth factor and can be a prognostic factor of treatment outcomes in CRC patients with oligometastatic disease.^26,30^ In our study, histological and molecular mutation for every treated site was not confirmed and thus KRAS mutation for every treated site is unknown and cannot be included in the analysis.

In conclusion, our study showed modest PFS, OS, and post-treatment toxicity outcomes which were comparable to the current published literature on SABR to oligometastases from CRC. It has highlighted that SABR potentially benefits small-sized tumours such as lymph nodes; and cumulative tumour volume may be a stronger prognostic factor of OS comparing to the number of metastases. Although the sample size of this study is small, this preliminary clinical hypothesis warrants further validation in a larger independent sample size under a multicentre randomised trial setting.
